# From Heart Health Promotion to Chronic Disease Prevention: Contributions of the Canadian Heart Health Initiative

**Published:** 2007-03-15

**Authors:** Kerry Robinson, Tracy Farmer, Susan J Elliott, John Eyles

**Affiliations:** School of Geography and Earth Sciences, McMaster University; Department of Anthropology, School of Geography and Earth Sciences, McMaster University, Hamilton, Ontario, Canada; School of Geography and Earth Sciences, McMaster University, Hamilton, Ontario, Canada; School of Geography and Earth Sciences, McMaster University, Hamilton, Ontario, Canada

## Abstract

**Background:**

The Canadian Heart Health Initiative began in 1987 as an 18-year undertaking to address the epidemic of cardiovascular disease in Canada. There is growing recognition in Canada of the need for an integrated approach to prevention that addresses common risks for many chronic diseases.

**Context:**

Research and intervention activities of the Canadian Heart Health Initiative have shifted toward chronic disease prevention and health promotion. This study explores the contributions of the Canadian Heart Health Initiative to document how single-disease strategies can evolve into integrated chronic disease prevention efforts.

**Methods:**

Key informant interviews were conducted with project researchers and health system stakeholders from seven Canadian Heart Health Initiative provincial projects. A review of provincial health policy documents was also performed.

**Consequences:**

Findings indicate that the Canadian Heart Health Initiative projects contributed to public health capacity development, including coalition and partnership building, and development of health knowledge and resource infrastructure. The Canadian Heart Health Initiative projects helped put chronic disease prevention issues onto local and provincial health agendas and provided community-based models to help develop public health policies.

**Interpretation:**

Experience with the Canadian Heart Health Initiative shows the need for integrated health programs to build on existing infrastructure. Other requirements for integrated chronic disease prevention programs include shared goals, partnerships at various policy levels and in multiple sectors, ongoing information sharing, and funding that is flexible and long-term.

## Background

Chronic diseases are often associated with common, modifiable risk factors (e.g., unhealthy diet, physical inactivity, tobacco use, alcohol overuse) and underlying social, economic, and environmental determinants (1). Most public health systems approach chronic disease prevention through fragmented prevention programs that are disease or risk factor specific (e.g., cancer prevention programs, tobacco reduction initiatives) (2,3).

Chronic disease prevention (CDP) and healthy living promotion (HLP) provide efficient, integrated approaches to multiple diseases and can use limited health resources effectively to improve program sustainability and reduce program duplication (4-6). CDP- and HLP-integrated approaches can address multiple risk factors and have potential to improve overall population health and to increase patient satisfaction (3). With the exception of information about the National Public Health Partnership, 2001 (7), our literature review found few examples of integrated CDP and HLP approaches to health programs and little documentation of their development or of the policies that support and impede them despite their potential contributions to public policy.

The term *integration* in CDP and HLP involves development of a unified policy framework that addresses common risk factors and social and environmental conditions for multiple diseases. Integration in this context means consolidating health promotion activities, combining population-based and high-risk strategies, building on existing prevention programs, employing multiple interventions, and engaging partners on issues that influence health (8). There is evidence that gradual policy shifts from a focus on single-disease prevention to more comprehensive, multiple chronic disease prevention are occurring.

An example of a multiple-disease prevention approach at the international level is the World Health Organization's CINDI (Countrywide Integrated Noncommunicable Diseases Intervention) program that links local and national level efforts in more than 30 countries to address common risk factors through collaborative programs (5). Another example is the Pan American Health Organization's CARMEN (Conjunto de Acciones para la Reducción Multifactorial de las Enfermedades No Transmisibles) initiative that combines preventive health services, health promotion initiatives, and policy work (9). Several individual countries, such as Singapore and Australia, have developed their own integrated prevention initiatives (10,11). Few empirical studies exist about the development of integrated CDP and HLP health strategies.

This study shows how strategies focused on a single disease can evolve and contribute to integrated CDP and HLP programs. We examine the dissemination phase of the Canadian Heart Health Initiative (CHHI) as a national example of how integration of CDP and HLP strategies can occur. We use a qualitative, multiple-case study to examine the integration of CDP and HLP in Canada. We consider CHHI contributions to this shift toward integrated chronic disease prevention and identify facilitators and barriers to an integrated strategy. This study is based on experiences of seven Canadian provincial projects in the CHHI dissemination phase.

## Context

In Canada, there are common interests and partners in the fight against chronic disease, but there are limited health promotion resources. This lack of resources has led to a policy shift toward integrated CDP and HLP programs at both national and provincial levels (12). This policy shift has been driven by national and provincial coalitions and alliances of civil, government, and professional organizations and has been informed by experiences of large-scale community disease prevention trials and disease-specific prevention initiatives (e.g., Stanford Five-City Project, North Karelia Project). The CHHI, a five-phase, 19-year undertaking during the period 1986 to 2005, addressed the cardiovascular disease epidemic in Canada. The CHHI, the Canadian Cancer Control Strategy, and the Canadian Diabetes Strategy are key Canadian disease prevention initiatives from this period. Although CHHI began as a national and provincial partnership to enhance heart health, its research and intervention activities have evolved over time and contributed to CDP and HLP efforts across Canada.

**Figure 1:**
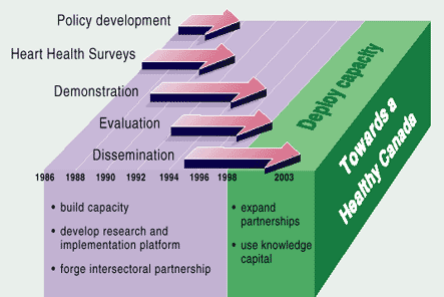
Phases of the Canadian Heart Health Initiative, 1986 to 2005.

Guiding principles of CHHI include 1) health program collaboration at national, provincial, and local levels; 2) recognition of the need to build health research and intervention capacity; 3) integration of programs into existing public health systems; 4) incorporation of population-based and high-risk approaches to programming; and 5) targeting of common chronic disease risk factors and interventions (13). From 1986 to 2003, CHHI evolved through stages of national policy development, risk factor surveys, demonstrations of programs and interventions, and health program evaluations and dissemination (Figure 1).

**Table:** 

The CHHI dissemination phase extended 1) best practices (e.g., coalition models, health programs) developed in the demonstration phase; 2) capacity-building interventions; and 3) chronic disease research initiatives to examine factors affecting capacity and dissemination of community-based health promotion (14).

## Methods

We received ethics clearance from the McMaster University Research Ethics Board and undertook a case study of seven provincial projects involved in the CHHI dissemination phase. Projects were located in Ontario from 1994 to 1998, Manitoba from 1996 to 2001, Prince Edward Island from 1996 to 2001, Saskatchewan from 1998 to 2003, Newfoundland and Labrador from 1998 to 2003, British Columbia from 1999 to 2004, and Alberta from 1999 to 2005. The seven projects focused on building capacity, disseminating heart health promotion innovations, and examining these processes over a 4- to 5-year period.

**Figure 2:**
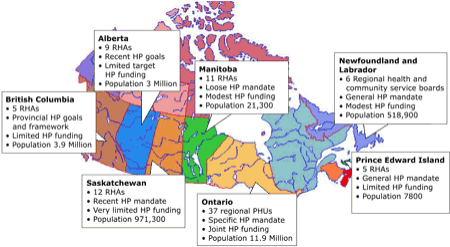
Canadian provinces participating in Canadian Heart Health Initiative (CHHI) dissemination phase study, 1994–2005. RHA indicates regional health authority; PHU, public health unit; HP, health promotion. Health system structures and population size reflect information at the time of the study. Dates vary by province. (See Methods section.) New Brunswick did not participate in the dissemination phase study. Quebec and Nova Scotia did not participate in the current study.

Because of differences in provincial health systems, the provincial projects targeted a diverse set of organizations (e.g., public health units, health districts, regional health authorities, community committees, coalitions) and used a variety of research designs (e.g., participatory action research case study, longitudinal mixed methods, qualitative parallel case study). The projects also occurred in varied geographic (rural, urban, mixed) and provincial health system contexts (Figure 2).

**Table:** 

These variations provided diverse provincial cases and reflected the diversity of the Canadian environments and organizations studied. Projects represented CHHI chronic disease prevention efforts with the exception of the Quebec project, which was not studied and which focuses on engaging medical professionals in screening and individual prevention through community health centers.

Assessment of project contributions is based on key informant interviews and analysis of recent provincial health policy documents. We sampled key informant interview respondents (12 to 15 respondents per province, n = 95) to achieve maximum response variation on a range of topics. Respondents included people who were project investigators, staff, stakeholders working at frontline and management levels in government, and those working at nongovernmental and community agencies. More than 50% of respondents were project stakeholders, and approximately 45% of respondents were project researchers. Respondents had an average of 3 years of involvement with their projects. Interviews were guided by a checklist that included 1) interventions and changes related to health promotion capacity building and dissemination, 2) research activities, 3) contributions to chronic disease prevention, 4) provincial context, and 5) facilitators and barriers to programs and interventions. All interviews were taped and transcribed verbatim by research assistants.

Analyses of respondent interviews were supplemented by a review of recent provincial chronic disease prevention and government health promotion policy documents (15-21). One document from each province was included (n = 7), and we examined contextual factors influencing governmental policy initiatives for the health system in each province in the areas of chronic disease prevention and promotion of healthy living.

Both interview transcripts and policy documents were imported into NUD*IST N5 (QSR International, Melbourne, Australia), a qualitative software for thematic analysis that uses coding to index, search, summarize, and analyze data (22). Analysis of provincial cases included searches for frequency of themes and patterns in the development of chronic disease programs to show similarities and differences among provinces. One subset of interviews (n = 10) and policy documents (n = 3) coded by two researchers showed approximately 70% agreement of detailed coding and indicated strong coding dependability (23). We validated the provincial analysis summaries through a member-checking process (24) by having interview respondents and project researchers review summary reports to determine accuracy.

## Consequences

### The shift in chronic disease prevention and promotion of healthy living approaches

The timing of the initiation of integrated chronic disease prevention and healthy-living promotion strategies varied by province, but all began during or shortly after their respective CHHI dissemination phase. Identification of the policy approach used, who the policy leaders were, and which implementation bodies were involved is shown in Table 1. In some cases, provincial policy movement toward CDP and HLP preceded and contributed to national policy development. In other examples, provincial policy followed national policy. Manitoba led in mobilization of integrated chronic disease prevention through the formation in 1997 of a partnership of health-related nongovernmental organizations (NGOs) focused on linking and promoting programs and advocacy for government funding to support comprehensive chronic disease prevention (25).

Since the Manitoba effort in 1997, other provincial governments (Newfoundland and Labrador, Prince Edward Island, British Columbia) and new NGO partner alliances (Alberta, Ontario, Saskatchewan) began their own policy shifts or were spurred on by national policy development efforts for the prevention of chronic diseases. Groups such as the Chronic Disease Prevention Alliance of Canada, a network of more than 50 national and provincial organizations, began developing policy strategies to address chronic diseases and related risks.

Differences in which group led provincial integrated approaches (government or NGO alliances) appear to be based on the affiliations of champions and which group had the political will to make CDP and HLP efforts. Ontario, Prince Edward Island, and British Columbia adopted a chronic disease prevention and healthy living approach focused on traditional multirisk factors. Newfoundland and Labrador, Alberta, and Saskatchewan embraced a broader health promotion and healthy living approach that recognized a wide range of health issues (e.g., mental health, injury prevention) from a population health perspective. There is a split in the provinces between those that target CDP and HLP implementation through regional health authorities (Saskatchewan, British Columbia, Alberta) and those that use regional coalitions and community committees made up of regional public health organizations; NGOs; and school, workplace, and citizen groups (Manitoba, Newfoundland and Labrador, Prince Edward Island, Ontario).

During their respective CHHI dissemination phase, all but two of the provinces studied (Ontario and Manitoba) shifted focus away from heart health promotion and toward chronic disease prevention and promotion of healthy living. All provinces that broadened their focus to include chronic disease prevention and promotion of healthy living did so based on feedback from regional health authorities and coalitions. These regional health organizations and coalitions indicated interest in a broadened approach to chronic disease prevention and showed that regional activities were already making a transition to integrated approaches to maximize resources and partnerships.

When we went out in 2000 to ask what they [regional health authorities] were doing here in heart health, they said, "Well, we're not just doing heart health — we're doing chronic disease prevention because we are working across the stove pipes. . . . We don't have the resources or capacity to just do heart health and cancer and diabetes separately. . . . We might have one person who is covering off all those areas." Alberta interview, 2005

The Ontario project did not make a shift to integration of chronic disease prevention during the CHHI dissemination phase because the project operated in the middle to late 1990s when chronic disease program integration was not a national or provincial focus. During the final year of the dissemination phase (1998), Ontario was the first province to fund multirisk-factor coalition programs from provincial resources. Since that year, Ontario has renewed this funding and relabeled it as *chronic disease prevention funds* to reflect the focus and use of the funds. Manitoba did not shift the focus of its technical assistance support but continued to operate with the understanding that multirisk-factor heart health efforts would influence other diseases, such as diabetes, that were prevalent in the province.

Respondents indicated that projects operating later in the CHHI dissemination phase (2000 to 2005) took place in a national and interprovincial policy context that encouraged integrated chronic disease prevention. This encouragement for chronic disease integration was different from the context 5 or 10 years previously, when individual disease strategies were the approach of the day.

I think even at the CHHI level you have to recognize that if you didn't get on the chronic disease bandwagon because of the risk factors, because of funding issues, political issues, reality issues, then you were missing the boat. . . . So yes, it did shift — it shifted from promoting heart health to chronic disease. British Columbia interview, 2004

### Contributions of the CHHI to Canadian integration of chronic disease prevention

Approximately 80% of interview respondents reported that CHHI dissemination projects made contributions to integration of chronic disease prevention in their provinces. Another 15% of respondents did not comment on CHHI project contributions to CDP and HLP, 4% noted they did not have adequate knowledge to comment, and one respondent stated that the respective provincial project did not have significant impact on CDP or HLP.

Respondents from Newfoundland and Labrador, Prince Edward Island, Saskatchewan, British Columbia, and Alberta stated that provincial projects broadened the focus of local capacity-building and dissemination interventions (e.g., training, networking, advocacy) from heart health to chronic disease prevention, healthy communities, and population health promotion. Interviews revealed that respondents identified three main areas in which provincial projects contributed to the shift toward chronic disease prevention and healthy living promotion: 1) knowledge and resource development, 2) coalition and partnership building, and 3) policy advocacy and strategy development (Table 2).

There was variation among respondents regarding the primary areas in which provincial projects contributed to integrated CDP and HLP approaches. Manitoba, Prince Edward Island, and Ontario were most often mentioned as contributing to knowledge development for CDP and HLP. Alberta and British Columbia were mentioned as contributing to coalition and partnership building, and Saskatchewan and Newfoundland and Labrador were mentioned as contributing to CDP and HLP knowledge, partnership building, and policy development.

#### Knowledge and resource development

More than two thirds of respondents identified knowledge of capacity-building processes (e.g., dimensions of capacity, development strategies, public health infrastructure needs) and development of health promotion knowledge among public health practitioners as key components of integrated chronic disease prevention strategy support. These key aspects are best attained through staff training and shared research results. Newfoundland and Labrador, Prince Edward Island, and Manitoba  contributed knowledge about integrated chronic disease prevention models and practices for community coalitions. This knowledge is now central to their provincial CDP and HLP chronic disease prevention strategies. Manitoba, Newfoundland and Labrador, Ontario, and Alberta established or supported development of resource centers and systems to support capacity building for CDP and HLP. In several cases, provincial projects developed resource centers and systems to provide technical assistance for chronic disease prevention where no supports previously existed. The Alberta, Newfoundland and Labrador, and Manitoba projects created positions for provincial-level program managers and technical assistance staff to facilitate material dissemination, consultations, and networking.

#### Coalition and partnership building

Forty-four percent of respondents reported that CHHI projects played a lead role in creating partnerships and alliances at multiple levels and in bringing together stakeholders (e.g., provincial NGOs, professional associations, government departments, public health organizations, social services, sports groups) to address CDP and HLP issues. The national CHHI network and investigator group facilitated interprovincial exchange of research and intervention activities. This exchange led to collaboration on national research, such as risk factor surveys, and to dissemination of research findings, interventions, and evaluations, such as the demonstration-site process evaluation. These exchanges provided a provincial information base linked to policy for CDP and HLP.

The Manitoba, British Columbia, Saskatchewan, and Alberta project teams stand out as being key champions in creation of provincial alliances and partnerships and in development of provincial CDP and HLP strategies. The Newfoundland and Labrador and Prince Edward Island teams played support roles in bringing government together with provincial partners to identify and address common interests. Ontario's research contributed to a provincewide program with governmental funding to support regional and local coalitions linked to public health systems. British Columbia facilitated regional and provincial networking and provided seed funding for formation of regional alliances. The Manitoba, Newfoundland and Labrador, and Prince Edward Island teams established and supported community coalitions that formed the basis for implementation of formalized CDP and HLP initiatives.

#### Policy advocacy and strategy development

More than one third of respondents reported that their provincial projects helped establish CDP and HLP issues on regional, provincial, and national policy agendas. Project champions from Newfoundland and Labrador and Manitoba led policy advocacy and development efforts for CDP and HLP in their provinces. These efforts contributed to provincewide chronic disease prevention and wellness initiatives (15,16) that attracted modest provincial funding and technical support in both provinces. The Saskatchewan, Prince Edward Island, British Columbia, and Alberta project teams either led or supported partnerships with others to develop provincewide strategies or policy frameworks for CDP and HLP. These strategies and policies helped guide regional efforts and provincial coordination of chronic disease prevention efforts (17-20).

Ontario's research findings and tools were used for development and evaluation of a provincewide multirisk-factor heart health program based on regional community partnerships and supported by both provincial funds and regional in-kind contributions (21). All seven provinces have released provincewide CDP and HLP policy strategies in the past 2 years, and several of these strategies have accompanying implementation and capacity support funds.

#### Facilitators and barriers to integrated chronic disease policy and action

Respondents spoke often of barriers to integrated provincial chronic disease policy and action (Table 3). The most commonly identified barrier was lack of financial resources and commitment by provincial governments for CDP and HLP strategies. This lack of funding and commitment was perceived to be related to competing priorities for policy attention and resource investment in acute care systems — areas considered to be in crisis by many provinces (26). Respondents spoke of a lack of political will to move forward on integrated CDP and HLP strategies in some provincial areas.

There are a lot of people out there who want it [chronic disease prevention], there are a lot of people out there who understand it. . . . It is just an uphill battle. This province is not about prevention at the present time. This province is about getting cost containment on those things that are driving medical services and hospital things right off the map. And it isn't about prevention; it is not even on the radar. British Columbia interview, 2004

Respondents indicated that the competitive nature of NGOs, such as the Heart and Stroke Foundation and Cancer Society, have translated into turf competitions, which at times compromise interagency partnerships on common risk factors and other issues. There are a number of process issues, such as the lengthy time it takes to develop consensus for provincial-level partnerships, that impede planning and implementation of CDP and HLP programs.

Some of the challenges are that CDP could encompass absolutely everything. So how big do you make it? How do you operationalize it at the community level? How do you make sure that it continues to be appealing and relevant to different stakeholders? How do you make sure that the public understands it? That is a really big message to give somebody. So I think there are a ton of challenges around it. Ontario interview, 2001

Respondents noted that some factors facilitated provincial planning and coordination efforts when diverse organizations worked on a joint CDP and HLP agenda. Dedicated champions were valued facilitators who used commitment to a broad vision to bring together different sectors and stakeholders to pool resources and expertise.

[We've had] some strong leadership provincially. The heart health team [members] have been good leaders linking the project to the Healthy Living Network. There is a strong person from Canadian Diabetes Association and also the Cancer Board. . . . The Alberta Public Health Association has been helpful. So a key group of individuals, including Medical Officers of Health [are needed]. So you need some people who are prepared to carry the flag in their own jurisdiction but then also to share and work together for integration as a provincial group. Alberta interview, 2005

NGOs and regional health organizations often have overlapping partnerships and find advantages in working together on common strategies because of limited resources and common risk factors. The research findings of CHHI provincial dissemination projects provided an evidence base for health promotion that has supported the case for CDP and HLP in the minds of policymakers. Increased public interest in health promotion coupled with pan-Canadian and national interest in integrated CDP and HLP policies provide support for provincial integrated health efforts to move forward.

I think the people are ready — there've been a number of indications. One is our benchmark survey we repeated just recently, and that indicates that the people clearly think that prevention and health promotion are important and that more resources need to go to it. . . . Certainly my experience in doing needs assessments in the community is that the people are very aware of what the health issues are and what the causes are and where we need an investment. Newfoundland and Labrador interview, 2003

## Interpretation

Public health strategies that target individual chronic diseases have historically operated without reference to one another. Their separateness has contributed to limited program effectiveness and efficiency (6,27). This study reveals strong commitment and collaboration as well as a knowledge base to support integrated CDP and HLP initiatives in Canada. Provincial dissemination projects and resulting collaboration have contributed to 1) integrated CDP and HLP policies, 2) combined research and intervention activities, 3) coalition and partnership building, 4) increased knowledge and resources, and 5) policy and strategy development. Some projects focused on partnership building, and others emphasized skill building and training.

CHHI dissemination project contributions align closely with strategies identified by the World Health Organization (5) as requirements to support integrated CDP and HLP initiatives: 1) multilevel partnerships, 2) policy development, 3) capacity building (e.g., knowledge and resource development), and 4) a combination of surveillance and information dissemination. CHHI projects have supported partner alliances between provincial governments and newly formed NGOs to develop CDP and HLP policy initiatives and have helped regional health authorities and coalitions implement health strategies at regional and community levels.

Respondents stated that the shift to an integrated chronic disease prevention strategy has been facilitated by provincial planning and coordination efforts and dedicated champions and leaders. It would be naive, however, to think that transition from a single-disease focus to a more comprehensive chronic disease prevention approach could occur without significant challenges. The key barriers identified by respondents as impeding provincial CDP and HLP policy action were lack of financial resources that span multiple-disease strategies and competing priorities (e.g., acute care and public health crises that divert policy attention and resources). These barriers were also identified by chronic disease prevention alliances in Canada as key challenges (28).

Other potential difficulties identified by respondents are confirmed by the literature and include the following: 1) issues for individual agencies of territoriality and perceived “loss of glory” (i.e., sharing credit for achievements) that may affect fundraising; 2) resource costs involved in creating partnerships and slow progress in making things happen; 3) problems integrating programs that have varied policies, service frameworks, and practices (i.e., silo effect); and 4) difficulty protecting underfunded programs when integrating them with programs that have adequate resources(29,7).

The Canadian experience with creating integrated CDP and HLP programs through the CHHI initiative provides information that may assist others. To overcome challenges inherent in integrated approaches to public health, it is important to 1) develop successful partnerships at multiple policy levels (e.g., national, provincial, regional) and 2) include government, NGO, and research organizations and programs. Sharing information and goals and coordinating efforts are critical to stimulating joint efforts, reducing duplication, and increasing the likelihood that goals will be achieved. Building on existing initiatives and partnerships helps ensure coherent services and improve program feasibility and sustainability. Financing integrated initiatives is critical and requires flexibility for provincial-, regional-, and community-level organizations to determine how funds should be allocated. The impact of public health policy and systems integration on chronic disease health outcomes takes time to occur, requires coordinated funding for CDP and HLP capacity development, and needs reliable strategy implementation over an extended period of time (10 years or more).

The different intervention paths and outcomes of the Canadian CHHI provincial projects show that no one approach works best. Each project had to adapt interventions to the local political and health system context. The Canadian story about integrated chronic disease prevention may hold insight for other jurisdictions, but most findings cannot be generalized directly. Countries have individualized health system structures and policy environments that will play a central role in shaping the evolution of integrated CDP and HLP programs worldwide.

### Epilogue

The Canadian government recently announced a plan to invest $300 million (CD) over 5 years (2006 to 2011) in the Integrated Strategy on Healthy Living and Chronic Disease. This strategy is based on capacity building, and advocacy efforts that originate from regions, provinces, and national policies. The design and implementation of this national strategy and the spin-off effects at provincial and regional levels will provide further opportunities for new insights and exchanges with other jurisdictions in the pursuit of an integrated approach to chronic disease prevention and healthy living promotion.

## Figures and Tables

**Table 1:** Chronic Disease Prevention and Healthy Living Promotion Programs, Canada, 1994–2004

Year of Project Initiation	Project Location	Type of Health Initiative	Policy Leaders	Implementation Groups
1997	Manitoba	Chronic disease prevention	Provincial NGO alliance	Community committees
2002	Newfoundland and Labrador	Wellness^a^	Government	Regional coalitions
2002	Alberta	Healthy Living^a^	Provincial network	Regional health authorities (RHAs)
2002	Prince Edward Island	Healthy Living^a^	Government	Regional coalitions
2003	Ontario	Multirisk factor and chronic disease prevention	Government and NGO alliance	Regional coalitions
2003	British Columbia	Healthy Living^a^	Government	RHAs and regional coalitions
2004	Saskatchewan	Population Health Promotion^a^	Government and intersectoral group	RHAs

NGO indicates nongovernmental organizations.

a Wellness, Healthy Living, and Population Health Promotion programs address a broad range of health issues (e.g., mental health, injury prevention), and specific issues vary by province.

**Table 2:** Canadian Heart Health Initiative (CHHI) Contributions and Quotes From Key Informant Interviews From Seven Provincial Projects, Canada, 1994–2004

CHHI Contribution	Illustrative Quotes About the CHHI Role in Integrated Chronic Disease Prevention and Healthy Living Promotion
Knowledge and resource development	The main contributions are the development of our community mobilization framework. . . . It's a very good model for how you enter a community, activate a community, organize, and make it sustainable. . . . We fleshed out how you actually do these various processes and how you share power, how you build capacity, how you create common vision and goals (Prince Edward Island interview, 2001). Our findings helped inform not just the local level but the central resource system [by] shaping the Ontario Heart Health Resource Centre and helping to create the idea of a coordinated set of resource centers. . . . It helped them create a knowledge-needs feedback loop between the central support resource structure and the frontline people (Ontario interview, 2001).
Coalition and partnership building	The CHHI national process and our ability to interact regularly helped . . . for integrating both research and intervention, and through that integration the heart health community stayed together and was morphing into chronic disease [prevention]. We are very connected to chronic disease because we were involved in heart health. . . which led to best practices work and the G8 [database] process. British Columbia interview, 2004). An outgrowth of that [Manitoba project] was the Alliance for the Prevention of Chronic Disease, and without being tuned into that, I think we wouldn't be as far as we are. Their ability now to develop a rapport with the people on the committees has enabled them to go on to working in partnership on a physical activity strategy, and the networking that has taken place provincially is a result of that significant shift in how they've operated, and that partnership has opened a lot of doors (Manitoba interview, 2001).
Policy advocacy and strategy development	The Heart Health program and its staff . . . kept us honest about focusing very upstream. The same thing related to the development of the Provincial Population Health Promotion Strategy. . . . Their message was always very strongly related to being upstream, focusing on determinants, ensuring that the community is engaged, and ensuring that other sectors engage. They've really pushed that envelope and advocated for that envelope as we embark on new initiatives or continue with others (Saskatchewan interview, 2003). We want to take these heart health coalitions and make them wellness coalitions that were written into our strategic health plan for the province. That speaks to the influence that they've had. There were a group of us who understood what the role of the heart health coalitions had been to date, and then we were able to . . . actually work with others to show them that this would be a really good mechanism for us to use to really move some of the provincial objectives forward (Newfoundland and Labrador interview, 2003). Definitely I can say that the Heart Health Initiative over the years has had an influence at the provincial level and at the regional level. Through their [heart health team's] central role in facilitating the Alberta Healthy Living Network . . . there is no question in Alberta that chronic disease prevention/healthy living is very high on the political and government agenda. The findings from the Heart Health Project have been carried through into other initiatives. As a result, we currently provide or recently provided significant funds on initiatives [through the heart health team] for pilots in three communities in the province all around healthy living capacity building (Alberta interview, 2005).

**Table 3:** Factors Cited by Provincial Respondents as Facilitators and Barriers to Integrated Chronic Disease Prevention and Healthy Living Promotion, Canada, 1994–2004

Facilitator or Barrier	No. Respondents Citing Factor as a Facilitator or Barrier, n (%) (N = 95)
**Facilitators**	
Strong provincial planning and progress	19 (20)
Dedicated champions for integration process	15 (16)
Public interest in health promotion	9 (10)
Recognition of need and organizational support for partnerships	9 (10)
Availability of research and information	9 (10)
Common risk factor agenda	6 (6)
Similar policy interests	3 (3)
**Barriers**	
Lack of financial resources	26 (27)
Competing organizational priorities	20 (21)
Competitive nature of NGOs	12 (13)
Frustration with process and progress of integrated programs	7 (7)
Lack of coordination and silo aspect of organizations	5 (5)
Turnover and lack of leadership	5 (5)
Integrated programs too diverse and diffused	3 (3)

NGO indicates nongovernmental agency.
