# Erratum, Vol. 4: Issue 2

**Published:** 2007-06-15

Because of an editing error, the references in the article "From Heart Health Promotion to Chronic Disease Prevention: Contributions of the Canadian Heart Health Initiative" were incorrectly presented. References (4) and (14) were omitted from the text of the article; one source (Creswell JW. Research design: qualitative, quantitative, and mixed methods approaches. 2^nd^ edition. Thousand Oaks (CA): Sage Publications; 2003) was omitted from the list of references; and several of the references were not numbered consecutively. Corrections were made on our Web site on June 15, 2007, and appear online at www.cdc.gov/pcd/issues/2007/apr/06_0076.htm. We regret any confusion or inconvenience this error may have caused.

