# Erratum, Vol. 4: Issue 2

**Published:** 2008-06-15

Figure 2 in the article "From Heart Health Promotion to Chronic Disease Prevention: Contributions of the Canadian Heart Health Initiative," which appeared in the April 2007 issue, was amended to indicate that the population value provided for each province is the value for the population studied within each province, not the value for the total population of the province. The changes were made on March 13, 2008, and appear online at www.cdc.gov/pcd/issues/2007/apr/06_0076.htm.

